# Intracranial Complication as a Manifestation of Clinical Onset in a Child With Insidious Ear Infection

**DOI:** 10.7759/cureus.26152

**Published:** 2022-06-21

**Authors:** Samuel Chu, Farhana A, Farid R

**Affiliations:** 1 Otolaryngology - Head and Neck Surgery, Hospital Shah Alam, Shah Alam, MYS

**Keywords:** pediatric ent, acute otitis media, intracranial abscess, complications of acute mastoiditis, otolaryngology-head and neck surgery

## Abstract

Acute mastoiditis is a common consequence of acute otitis media and may lead to intracranial complications. Common clinical presentations include otological complaints (i.e., otorrhea, otalgia, fever). Intracranial complication remains a rare manifestation of middle ear infection. We present the case of a child who presented with non-specific symptoms without any otological complaints. Prompt clinical assessment and imaging revealed an otogenic brain abscess with concurrent mastoiditis. Management of this child required both medical and surgical treatment by a multidisciplinary team.

## Introduction

Acute mastoiditis is a serious bacterial infection of the mastoid bone, which occurs because of acute otitis media [1]. Rarely, this can cause further complications, resulting in devastating extratemporal and intracranial complications, with significant morbidity and mortality.

Common clinical presentation includes otological complaints (i.e., otorrhea, otalgia, and fever). Intracranial complications remain a rare clinical manifestation of middle ear infections. Here, we describe the case of a six-month-old infant with an otogenic brain abscess as a result of insidious acute mastoiditis.

## Case presentation

We present the case of a six-month-old child who was born at term via cesarean section at the mother’s request and was otherwise well throughout the neonatal period. He was brought to the emergency department with the following signs and symptoms: fever, irritability, vomiting, and reduced oral intake. The fever had started six days before, and the remaining symptoms had started a few hours before presenting to the emergency department.

A pediatric consultation was sought, and a diagnosis of presumed meningitis was made based on the patient’s clinical features. During admission, the child’s mother denied any otological symptoms (i.e., otorrhea, otalgia, and mastoid tenderness). Upon assessment, the child was lethargic, breathing comfortably under room air, with oxygen saturation levels of 99%, afebrile, and no significant findings on cardiopulmonary examination. His blood pressure was 104/67 mmHg and his heart rate was 171 beats per minute. The child did not have facial asymmetry. Empirical antibiotic treatment was initiated with intravenous ceftriaxone 400 mg at 12 hours, fluid resuscitation, and symptomatic treatment. Further examination revealed that the child had macrocephaly without bulging of posterior fontanelle; hence, computed tomography (CT) of the brain was performed.

**Figure 1:**
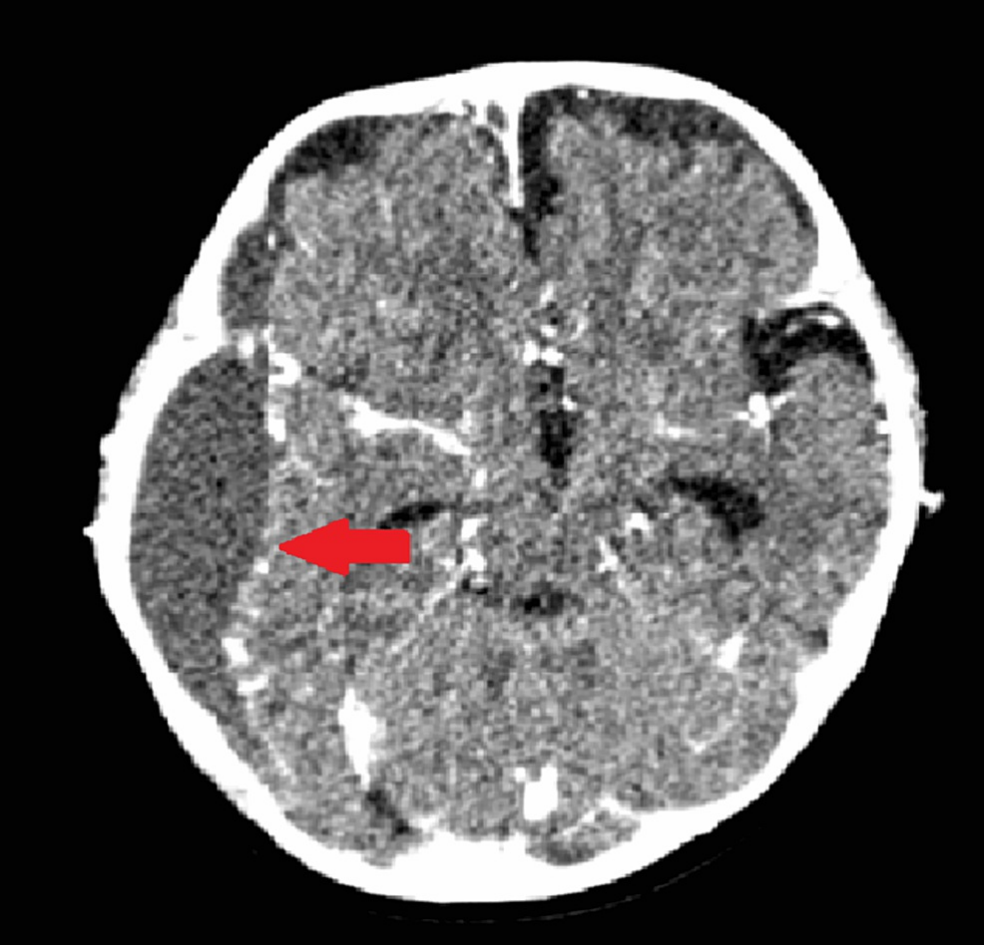
CECT brain axial view (parenchymal window) showing large right frontotemporal extradural empyema (measuring 8.9 × 2.4 × 9.2 cm) causing mass effect and midline shift. CECT: contrast-enhanced computed tomography

**Figure 2:**
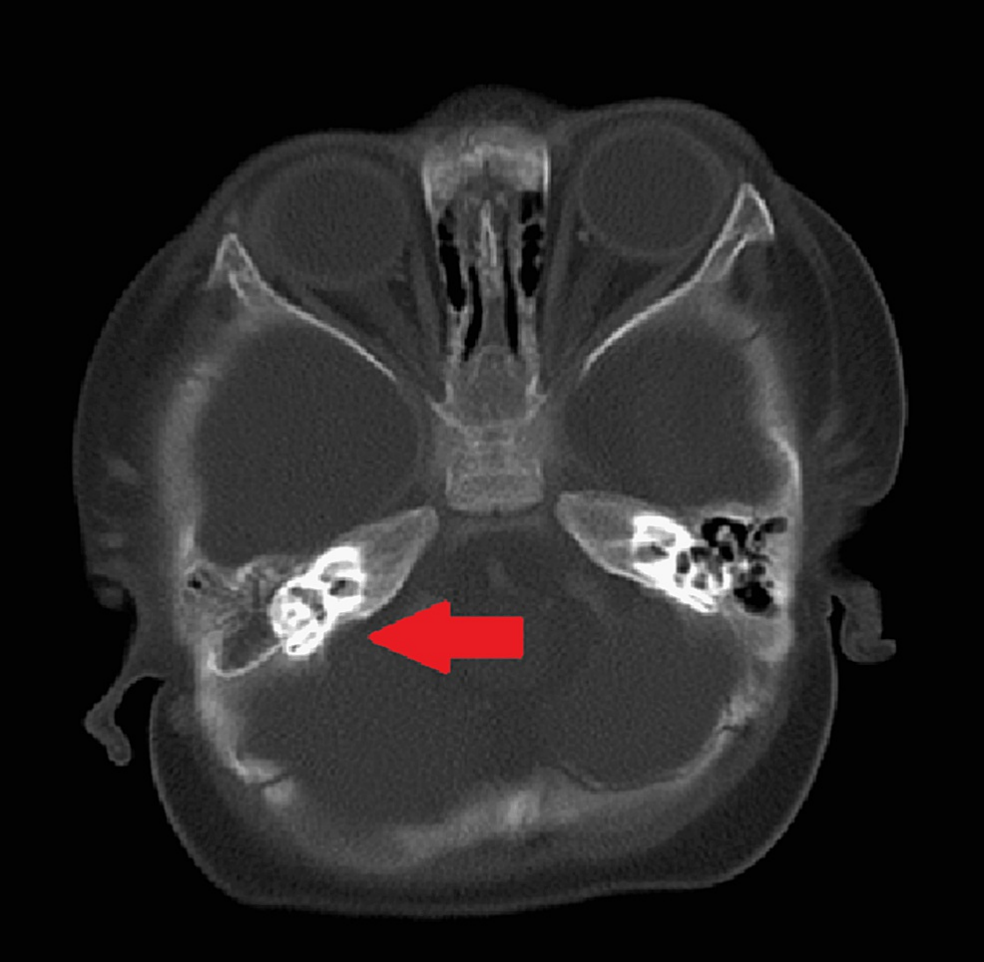
CECT brain axial view (bone window) showing fluid in the right mastoid air cells and middle ear cavity. CECT: contrast-enhanced computed tomography

Contrast-enhanced computed tomography (CECT) of the brain was performed on the second day after admission. As shown in Figure 1, a large right frontotemporal extradural empyema (measuring 8.9 × 2.4 × 9.2 cm) caused a mass effect and a midline shift. Incidentally, as shown in Figure 2, fluid was seen in the right mastoid air cells and middle ear cavity, representing mastoiditis and otitis media with the erosion of the tegmen tympani, raising suspicion of otogenic brain abscess. ENT and neurosurgical referrals were promptly performed by the pediatric team.

Our review of the child revealed that the child had a normal pinna appearance with no erythema or swelling in the retroauricular region. Otoscopy showed a mildly inflamed tympanic membrane; otherwise, his external auditory canal had a normal appearance. No posterior external auditory canal wall sagging, ear discharge, or granulation tissue were observed. Based on the clinical and CECT findings, we established the indication for surgery, and a right cortical mastoidectomy was performed. Under the same settings, the neurosurgical team performed a mini-craniotomy and drainage of the empyema.

Intraoperatively, 20 cc of pus was drained from the cranial cavity. Granulation tissue was observed in the mastoid air cells and the antrum. The tegmen tympani was thinned and covered with granulation tissue. However, no pus or effusion was observed in the mastoid region, middle ear, or the antrum.

On the third day of admission, a plain CT brain scan was repeated, showing a significantly smaller right frontotemporal subdural collection with improved mass effect and midline shift.

On the fifth day of hospitalization and treatment, the child developed a cough and experienced an episode of desaturation requiring intubation for impending respiratory collapse, and intensive unit care for one day. A diagnosis of nosocomial pneumonia was made based on the clinical findings and chest radiography. The antibiotic regimen was escalated to intravenous meropenem 350 mg TDS for eight weeks. The child was successfully weaned off oxygen on day 23 after admission. The patient was discharged home well, 73 days after admission, after completion of antibiotics. An automated auditory brainstem response assessment done later showed normal findings.

## Discussion

Acute mastoiditis remains the most common complication of acute otitis media, affecting one in 400 cases (0.24%) [2]. Its incidence ranges from 1.2 to 1.6 per 100,000 children aged 0-14 years, per year, varying in different countries and age groups. Given that the middle ear communicates with the mastoid area, involvement of the mastoid in infectious diseases of the middle ear is common. Intracranial complications occur in 4-16% of cases [3].

The main pathophysiology is represented by the closure of the aditus ad antrum due to edema or granulation tissue, which results in failure to drain purulent exudates from the mastoid air cells. Infection of the mastoid air cells with osteomyelitis may sometimes progress through the periosteum and induce periostitis and subsequent involvement of the surrounding structures, resulting in devastating extratemporal and intracranial complications with significant morbidity and mortality.

The signs and symptoms of acute mastoiditis generally do not differ from those of acute otitis media (otorrhea, otalgia, and fever). However, symptoms are usually more serious with pain over the mastoid area and sometimes with retroauricular erythema and swelling, even in cases where there is no purulent collection, and the cortical bone is not completely eroded.

The pediatric age group is most prone to mastoid involvement in acute otitis media, most commonly in the first year of life [4]. In children, the mastoid bone is more pneumatized with thin bone trabeculae, and the aditus ad antrum is smaller than that in adults. This predisposes children to the accumulation of secretions. Furthermore, the physiological immaturity of a child’s immune system contributes to disease progression. In summary, the disease evolves more rapidly and presents with more serious symptoms than in adults.

However, intracranial complications remain a rare manifestation of the clinical onset of middle ear and mastoid suppurations. In 2013, the Institute of Phonoaudiology and Functional ENT Surgery “Prof. Dr. D. Hociota,” Bucharest recorded a total of seven patients with insidious ear pathology presenting with intracranial complications [5]. The most common intracranial complications were meningitis, lateral sinus thrombosis, cerebellitis, and cerebrospinal fluid fistulas. In the same year, no cases of otogenic brain abscesses were reported.

In this case, the ear lesion evolved rapidly and was destructive to intracranial structures. The patient had no clinical manifestations until the occurrence of intracranial complications.

Fortunately, in this case, collaboration among specialists in pediatric, infectious disease, neurosurgery, imaging, and ENT led to satisfactory outcomes and remission of intracranial complications.

## Conclusions

Middle ear pathology can evolve rapidly without any otological symptoms and can manifest clinically only in the form of intracranial complications. Therefore, any signs and symptoms of ear pathology require prompt treatment and recognition of complications to avoid significant morbidity and mortality in children. Physicians must maintain a high level of clinical suspicion and perform thorough clinical (including neurological) examinations.
